# Measures of self-regulation used in adult rehabilitation populations: A systematic review and content screening

**DOI:** 10.1177/02692155221091510

**Published:** 2022-04-27

**Authors:** T.I. Mol, C.A.M. van Bennekom, E.W.M. Scholten, M.W.M. Post

**Affiliations:** 1 Center of Excellence for Rehabilitation Medicine, 526115 UMC Utrecht Brain Center, University Medical Center Utrecht, and De Hoogstraat Rehabilitation, Utrecht, the Netherlands; 2 84792 Department of Rehabilitation Medicine, University of Groningen, University Medical Center Groningen, Groningen, the Netherlands; 3 100506 Heliomare Rehabilitation Center, Research and Development, Wijk aan Zee, the Netherlands; 4 522567 Amsterdam University Medical Center, University of Amsterdam, Coronel Institute of Occupational Health, Amsterdam, the Netherlands

**Keywords:** Self-regulation, rehabilitation, patient reported outcome measures, systematic review

## Abstract

**Objective:**

We aimed to identify generic measures of self-regulation and to examine the degree to which these measures fit a recently developed conceptual model of self-regulation in a rehabilitation context.

**Data sources:**

Pubmed, Embase, PsycInfo, and CINAHL were searched.

**Review methods:**

Articles were included if they were published between January 2015 and August 2020 and reported on empirical studies (trials and observational studies) using a measure of self-regulation or a related concept, in an adult rehabilitation population. Main content was analysed by linking all items of the selected measures to one or more of the six sub-themes of self-regulation: (1) insight into physical and cognitive impairments, (2) insight into the consequences of the impairments, (3) insight into abilities, (4) to be able to communicate limitations, (5) trust in body and functioning, and (6) make use of abilities.

**Results:**

Two reviewers independently screened 7808 abstracts, resulting in the inclusion of 236 articles. In these articles, 80 different measures were used to assess self-regulation or related concept. Nineteen of these measures met the inclusion criteria and were included for the content analyses. Nine of these were self-efficacy measures. No measures covered four or more of the six sub-themes of self-regulation. The three sub-themes on gaining insights were covered less compared to the sub-domains ‘trust’ and ‘make use of abilities’.

**Conclusions:**

Many measures on self-regulation exist None of these measures cover all six sub-themes of self-regulation considered important to measure self-regulation as a rehabilitation outcome.

## Introduction

Persons living with a chronic health condition have to adapt to physical as well as psychological changes in their bodies and their lives. Medical rehabilitation treatment contributes to this adaptation and helps preventing, reducing and eliminating limitations caused by this health condition.^1,2^ Research has shown that effective rehabilitation consists of two types of intervention: (1) exercise, and (2) self-management and education.^1^ Interventions in self-management and education include gaining knowledge about the condition, about consequences of this condition, and learning skills in order to deal with them.^1^ The overall aim of rehabilitation is to improve a person's self-regulation, societal participation, and health-related quality of life (HRQoL).^3^

Self-regulation is a complex concept with diverging definitions and meanings for different target populations.^4–7^ Based on a qualitative investigation using seven focus group discussions among 40 former rehabilitation patients with various health conditions, we defined self-regulation as: “to create insights and awareness in own condition, limitations and possibilities, and give direction to your own life on all domains”.^8^ Also, we developed a comprehensive model of self-regulation for a rehabilitation population, based on perspectives obtained throughout these focus group discussions.^8^ Six subthemes were identified as important to learn or regain self-regulation during a rehabilitation trajectory. First, gaining self-insight was considered as a requirement to regain self-regulation. Self-insight was mentioned as crucial regarding; (1) physical and cognitive impairments (gaining a realistic view on the diagnosis itself), (2) the consequences of these impairments in terms of limitations (i.e. tiredness), and (3) the abilities. Second, it deemed important to learn how to deal with the consequences of impairment. Important learning sub-themes were mentioned as (4) to be able to communicate limitations, and (5) to have trust in body and functioning. Lastly, (6) making use of abilities, to optimise functioning in daily life, was mentioned as a final step in the process of regaining self-regulation. This conceptual model is further explained in the method section.

Measurement of important outcomes of rehabilitation can help to improve quality of care and to identify best practices, to monitor patients progress and is important for clinical research.^9,10^ A measure to evaluate self-regulation outcomes of rehabilitation should be in line with the definition and cover these six subthemes judged important by former patients.^11,12^ Several previous reviews were identified which provided overviews of measures that assess self-regulation, or related concepts.^13,14^ These reviews showed that most identified measures are either condition specific measures, such as the stroke self-efficacy scale, or task specific measures, such as the self-care self-efficacy scale. A few generic measures were identified, however they failed to report on test-retest reliability and validity parameters and therefore not widely used.^15,16^ Further, it is unknown to what extent existing generic measures cover the six subthemes from our conceptual model of self-regulation for a rehabilitation populations. One review was identified in which they conducted content analyses of generic measures. However, content analyses in this study was based on a different conceptual model which was not focussed on rehabilitation populations.^17^

We aimed to systematically review the literature to identify generic measures of self-regulation used in recent articles and examine the degree to which these measures cover the six subthemes deemed important to measure self-regulation as a rehabilitation outcome. Our research questions were:

1. Which generic measures were used to measure self-regulation, or related concepts, in an adult rehabilitation population in articles published between January 2015 and August 2020?
2. Does the content of these measures cover the six subthemes of self-regulation relevant in the context of medical rehabilitation, and if so, what are the clinimetric properties of these measures?

## Material and methods

We followed the steps recommended in the Consensus-based Standards of the selection of health Measurement Instruments (COSMIN)^18^ methodology for Patient-Reported Outcome Measures (PROMs) systematic reviews. We applied the Preferred Reporting Items for Systematic review and Meta-analysis (PRISMA) statement for reporting systematic reviews.

First, we searched the databases Pubmed, Embase, PsycINFO, and CINAHL for articles published between the 1^st^ of January 2015 and the 11^th^ of August 2020. We have chosen this timeframe aiming to include measures that were recently developed or still in use. We reasoned that older measures that were not used in any study published in the previous six years, apparently are considered less useful by the research community. The search string entailed four components:

1. The construct self-regulation and directly related concepts;
2. Patient Reported Outcome Measure (PROM) specifications;
3. The target population;
4. Exclusion of non-original research.

Details of the search strings for each database are shown in the online Supplementary file. By composing the search strings, most inclusion and exclusion criteria for the selection of the articles were set.

1. We identified related concepts by a preliminary literature search for concepts that were used in combination with self-regulation, such as self-efficacy, empowerment, self-concept, self-determination and self-control (all concepts are displayed in the online Supplementary file). Terms that are used in the context of care, such as patient-advocacy and self-care, were not considered to be closely related to self-regulation as defined in our study and were therefore excluded. Also, measures focussing on concepts different from self-regulation, such as psychological wellbeing, losing weight, or driving skills were excluded. Besides, we only included publications if generic measures of self-regulation or related concepts were used. Diagnosis-specific measures such as the ‘Multiple Sclerosis Self-Efficacy Scale’^19^ were excluded.
2. Inclusion criteria for the type of instrument, Patient-Reported outcome measure, was based on a standardised search filter. This filter has a sensitivity of 97.4% and a precision of 4.4%.^20^
3. We defined the target population as persons who had a diagnosis covered by one of the main diagnostic groups in medical rehabilitation in the Netherlands: (1) amputation, (2) neurological diseases (including neuromuscular diseases), (3) chronic pain disorder, (4) musculoskeletal disorder, (5) spinal cord injury, (6) acquired brain injury, or (7) organ disease or injury.^21^ We added ‘oncology’ as a diagnostic group due to the increasing number of patients with cancer in medical rehabilitation.^22^ Articles were included if the study focussed on one or more of these defined diagnostic groups. Articles that focussed on other diagnoses, such as autism, schizophrenia, stress-disorders, or Alzheimer's disease, non-patient target populations, such as caregivers or professionals, family members, military or abuse, were excluded. Further, this study focussed on an adult population. We included publications if at least 95% of the study population was 18 years of age or older.
4. For the exclusion filter also the standardised filter from the COSMIN was used.^20^

Lastly, we added to the search string that we restricted the search to articles that were published in the English language in scientific journals.

We merged all records into one file using the Reference management program Mendeley and uploaded this file in Rayyan QCRI,^23^ a systematic literature review web application. We removed all duplicates. In Rayyan QCRI the first author reviewed all records for inclusion, based on title and abstract. A random sample of 10% were independently screened by a research assistant. For screening of the records and articles, some additional exclusion criteria were set. We excluded qualitative research, reviews, study protocols, and validation studies. Records without an abstract were also excluded. The next step was the retrieval and review of the full-text articles to identify measures of self-regulation or related concepts, which was performed by the first author. A random sample of 10% were independently screened by the third author. We discussed all disagreements until consensus was reached. High levels of interrater agreement were found: 98.3% agreement (Kappa .92) in the title- and abstract screening, and 95.7% agreement (Kappa .91) in the full-text screening. Therefore we considered this 10% check to be sufficient. This review resulted in a list of measures used in one or more of the included studies.

For the review of these measures, we searched the internet to retrieve all measures. If we could not find a measure and did not receive a response after contacting the authors, we excluded the measure. Measures which turned out to be diagnosis-specific measures or duplicates used under another name were also excluded. From the measures which met all inclusion criteria, we extracted data on the following characteristics: author, year, number of articles in which the measure was used, construct as described by the author, number of items, sample item, sub-scales, response categories, score range (min-max), and interpretation and conditions for use.

Next, the content of the included measures was analysed by linking each item to the sub-themes of the previously developed conceptual model of self-regulation.^8^

The six subthemes are;

1. *To have insight into physical and cognitive impairments*. This subtheme focuses on the individual's understanding of their condition itself. In other words, does somebody have a realistic view on what this diagnose is and which signs and symptoms come with it. For example loss of sense or paralysis is due to spinal cord injury.
2. *To have insight into the consequences of these impairments.* This subtheme describes the understanding of the restrictions which come with the condition, such as tiredness, a decreased energy level, or having to use a wheelchair.
3. *To have insight into abilities.* This subtheme focuses on what is still possible for somebody, and to look for potential opportunities.
4. *To be able to communicate limitations.* This subtheme focuses on communication with other people about the condition and the resulting limitations, to relatives and individuals in their environment, to create an understanding of the situation for them as well.
5. *To have trust in body and functioning.* This subtheme focuses on having or having regained trust in one's own body and mind, in the newly discovered self, after a period of uncertainty due to the onset of a condition.
6. *To make use of abilities*. This subtheme focuses on optimisation of a persons’ functioning in terms of daily activities. That somebody does what he or she wants to do. It is also about own decision-making.^8^

If the content of one or more items of an included measure fitted the description of a sub-theme, that sub-theme was marked as covered by the measure. To compensate for the subjectivity in this screening, the third author screened 50% of the items as double-check and 92.1% agreement was found. We discussed disagreements and doubts with all four authors until consensus was reached. Before the screening, we specified the criterion that measures that covered four or more of the sub-themes, were considered eligible for clinimetric evaluation. However, as described below, no measures were found that covered four or more of the sub-themes of self-regulation and therefore we did not perform any clinimetric evaluation.

## Results

**Figure 1.**
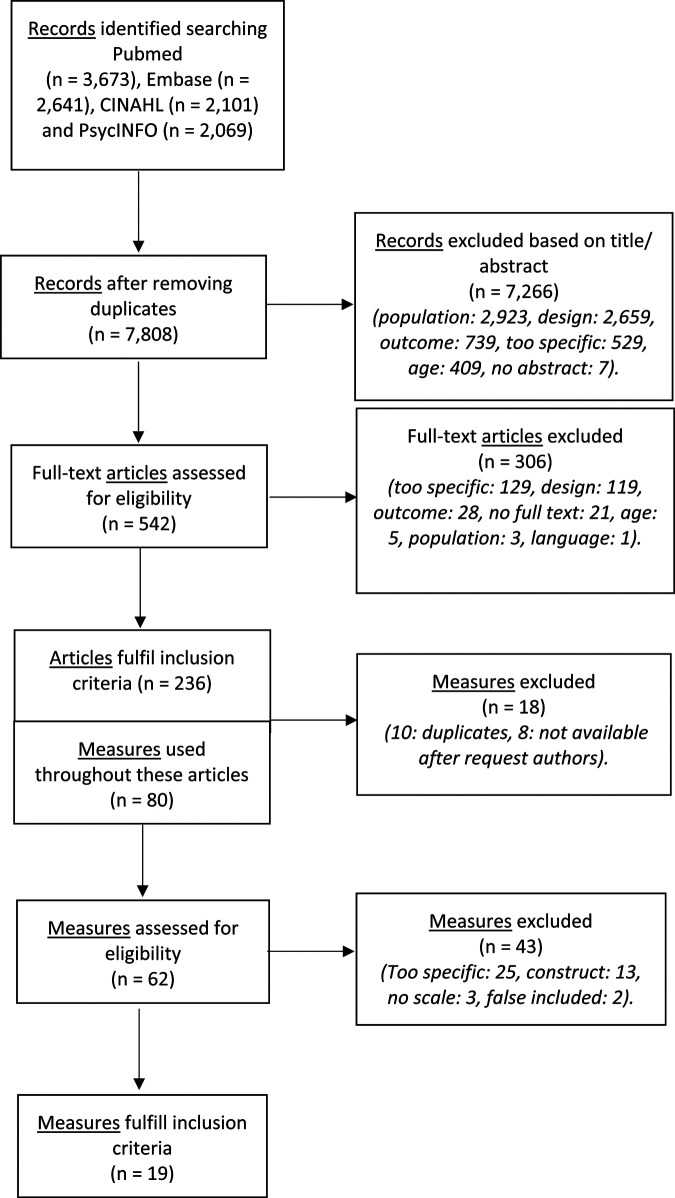
Flow diagram: identification of eligible measures for content analyses.

The search identified a total of 10,484 records. After removing duplicates, we screened the titles and abstracts of 7808 records. Most records were excluded because the study was performed in another population than we defined for this study, such as caregivers, or healthy subjects. Another main reason for exclusion was that the study did not use any measure. The full screening process is displayed in Figure 1. The screening resulted in a selection of 542 articles and we retrieved the respective full-text articles. After screening of the full text articles, a selection of 236 articles were identified. These 236 articles reported the use of 80 different measures for self-regulation or directly related concepts. Study populations included amputation (n = 7), neurological diseases (n = 43), chronic pain disorder (n = 9), musculoskeletal disorder (n = 24), spinal cord injury (n = 21), acquired brain injury (n = 47), organ diseases (n = 27), oncology (n = 34), and mixed diagnoses/ disabilities (n = 24).

Of all 80 measures, we found 19 measures eligible for content analysis. Reasons for exclusion at this stage were if measures turned out to be duplicates used under different names.^24–31^ For example the ‘chronic self-efficacy scale’ turned out to be the same measure as the ‘self-efficacy for managing chronic disease’.^24^ In such cases we merged the results and used the official name of the measure. Measures were also excluded if the full-text measure was not available,^32–39^ or if the measure was developed for use in a specific diagnosis or life domain.^25,40–63^ For example the ‘Moorong self-efficacy scale’^21^ was developed for use in persons with spinal cord injury and the ‘self-efficacy for rehabilitation outcome scale’^50^ was designed to measure physical improvement after rehabilitation. Further, measures which seemed to measure another construct than included in the search string were excluded.^28,31,35,64–73^ An example is the ‘perceived deficits scale’ which measures cognitive impairments.^29^ Measures were further excluded if the measure turned out to be no standardised measure, but an interview.^74–76^ Lastly, two measures were excluded that should have been excluded earlier in the process for example due to a different target population than we stated for this study.^77,78^ Details of all excluded measures can be found in Appendix 1.

Remarkably, seven measures which had ‘self-regulation’ in their name, were excluded at this stage. Four measures were too specific. focussing either on physical exercise or on psychological wellbeing/ mood.^47,51,52,79^ The other three were not available,^33^ measured a different outcome than we defined for this study (weight loss),^65^ or turned out to be not a measure.^75^

Table 1 reports general information on the nineteen included measures.^24,26,29,80–94^ Eleven measures were used in just one publication. Eight measures were used in two publications or more. The General Self-Efficacy Scale^88^ was used the most, with 91 publications reporting the use of this measure. The majority of the included measures focussed on the construct of self-efficacy.

**Table 1.** Included measures after screening.

Instrument (Authors: Year)	In how many publications included in this screening the measure was used	Construct based on author's perspective		N of items	Question example	Sub-scales	Response categories	Score (min-max) + interpretation	Conditions for use
General self-efficacy scale (Schwarzer & Jerusalem)^88^	91	Self-efficacy		10	I can always manage to solve difficult problems if I try hard enough.	-	4-point scale (Not at all true – exactly true)	10–40 (Higher score = better self-efficacy)	Free
Self-efficacy for managing chronic disease 6-item scale (Lorig & Ritter)^24^	19	Self-management		6	How confident do you feel that you can keep the fatigue caused by your disease from interfering with the things you want to do?	-	10-point scale (Not at all confident – totally confident)	1–10 (Higher score = better self-efficacy)	Free
Awareness questionnaire (Sherer)^89^	6	Awareness of deficits		17	How good is your ability to live independently now as compared to before your injury?	-	5-point scale (worse than before – total better than before / totally – totally not).	17–85 (Higher score = better awareness)	Free
University of Washington self-efficacy scale (Amtmann)^29^	6	Self-efficacy		19 (6 items in short version)	You can keep the physical discomfort related to your health condition or disability from interfering with the things you want to do?	-	5-point scale (not at all- completely)	10–90 (Higher score = better self-efficacy)	Free
Self-efficacy scale (Sherer et al.)^87^	4	Self-efficacy		23	I am a self-reliant person.	2: *General self-efficacy. *Social self-efficacy.	5-point scale (strongly disagree – strongly agree)	23–230 (Higher score = better self-efficacy)	Free with permission
Coping self-efficacy scale (Chesney)^90^	4	Self-efficacy		26	I am confident that I can talk positively about myself.	-	11-point scale (cannot do at all – certain can do)	10–260 (Higher score = better self-management)	Free
Health education impact questionnaire (Osborne & Elsworth)^91^	3	Self-management		42	When I have symptoms, I have the skills to cope.	8: *The positive and active engagement in life. *Health directed behaviour. *Skill and technique acquisition. *Constructive attitudes and approaches. *Self-monitoring and insight. *Health services navigation. *Social integration and support. *Emotional wellbeing.	4-point scale (Totally not agree – Totally agree)	1–4 (Higher score = better self-management)	Free with permission
Participation strategies self-efficacy scale (Lee et al.)^92^	1	Self-efficacy		37	How confident are you that you can strategize fatigue and find ways to save energy.	5: *Managing home participation. *Planning and managing community participation. *Managing work and productivity. *Advocating for resources. *Communication management.	10-point scale (not at all confident – total confident)	Sum of item scores per domain scale (range depends on number of items within the subdomain)	Free with permission
Personal advocacy activity scale (Hawley et al.)^93^	1	Self-advocacy		12	In the past 3 months … how many times you have negotiated with someone to get your needs met.	-	3-point scale (Not at all – 1–4 times – 5 or more times)	12– 36 (Higher score = better personal advocacy)	Free
Decision self-efficacy scale (O’Connor: 1995)^26^	2	Confidence		11	I feel confident that I can ask for advice.	-	5-point scale (Not at all confident – very confident)	0–100 (Higher score = better self-efficacy)	Free
Liverpool self-efficacy scale (Airlie et al.)^94^	1	Self-efficacy		11	Sometimes I feel that my […] controls my life.	2: *Control. *Personal agency.	4-point scale (strongly agree – strongly disagree)	Sum of item scores per domain scale (range depends on the number of items within that scale).	Free
Self-perception scale (Chen)^80^	1	Self-perception		8	Do you accept your present physical state?	3: *Confronting difficulties. *Self-value and confidence. *Opportunities and restrictions.	5-point scale (strongly refuse – strongly accept)	8–40 (Higher score = better self-perception)	Free with permission
Disability centrality scale (Bishop & Allen)^81^	1	Quality of live & control		4 questions: 10 domains (total of 40 items)	How much control do you have over changing this part of your life?	10: *Physical health. *Mental health. *Work (or study). *Leisure activity. *Financial situation. *Relationship with your spouse. *Family relations. *Other social relations. *Autonomy/independence. *Religious/spiritual expression.	7-point scale (Not very – very important/ control/ satisfied/ impact)	4–28 per domain scale (Higher score = better control)	Free with permission
Daily living self-efficacy scale (Maujean)^82^	1	Self-efficacy		12	Take part in new hobbies and new activities.	3: *Activities of daily living. *Psychological. *Social.	0 – 100 (Cannot do at all – highly certain can do)	0–100 (Higher score = better self-efficacy)	Free with permission
The self-advocacy scale (Hawley et al.)^93^	1	Self-advocacy	8		I can keep track of important information that I need.	-	4-point scale (Not confident - very confident)	8–32 (Higher score = better self-advocacy)	Free
Patient self-advocacy scale (Brashers & Kingle)^83^	1	Psychological autonomy	12		I actively seek out information on my illnesses.	3 *Illness education. *Assertiveness. *Mindful non adherence.	5-point scale (strongly agree – strongly disagree)	1–5 (Higher score = better autonomy)	Free
Self-determination scale (Sheldon)^85^	1	Self-awareness and own choice	10		A. I always feel like I choose the things I do. B. I sometimes feel that it's not really me choosing the things I do.	2 *Awareness of self. *Perceived choice.	5-point scale (Only A feel true – only B feels true)	5–25 (Higher score = better self-awareness/ own choice)	Free
Rosenbaum's self-control scale (Rosenbaum)^86^	1	self-control	20		When I act before I think, I tell myself to stop and think before I do anything.	-	10-point scale (Not true about me – true about me)	20–200 (Higher score = better level of enabling skills)	Free
PROMIS general self-efficacy scale (Gruber-Baldini et al.)^84^	1	Self-efficacy	10		It is easy for me to stick to my aims and accomplish my goals.	-	5-point scale (I am not at all confident – I am very confident)	1–5 (Higher score = better self-efficacy)	Free with permission

The nineteen included measures were linked to the six sub-themes of the self-regulation model. Table 2 shows per measure the number of items that could be linked to the six self-regulation sub-themes. Most measures included items that could not be linked to one of these self-regulation sub-themes, for example: ‘make unpleasant thoughts go away’^90^ and ‘use strategies to find ways easier in your home’.^92^ These items were too broad or dealing with different topics.

**Table 2.** Content analyses: The number of items per measuring fitting with the sub-themes.

Name of the measure	Sub-theme 1: *To have insight into physical and cognitive impairments*	Sub-theme 2: *To have insight into the consequences of these impairments*	Sub-theme 3: *To have insight into abilities*	Sub-theme 4: *To be able to communicate limitations*	Sub-theme 5: *To have trust in body and functioning*	Sub-theme 6: *To make use of abilities*	Items not fitting one of the sub-themes / total N of items	N of sub-themes in the measure
General self-efficacy Scale^88^	0	0	0	0	3	7	0/10	2
Self-efficacy for managing chronic disease 6-item scale^24^	0	0	0	0	6	0	0/6	1
Awareness questionnaire^89^	4	8	3	0	0	0	2/17	3
University of Washington self-efficacy scale^29^	0	0	0	0	17	0	2/19	1
Self-efficacy scale^87^	0	0	0	0	3	3	17/23	2
Coping self-efficacy Scale^90^	0	0	2	0	11	1	12/26	3
Health education impact questionnaire^91^	6	0	0	0	6	10	20/42	3
Participation strategies self-efficacy Scale^92^	0	0	5	5	0	6	21/37	3
Personal advocacy activity scale^93^	1	0	0	2	0	0	9/12	2
Decision self-efficacy Scale^26^	0	0	1	0	2	0	8/11	2
Liverpool self-efficacy Scale^94^	1	0	0	0	5	1	4 /11	3
Self-perception Scale^80^	0	0	1	0	2	0	5/8	2
Disability centrality Scale^81^	0	1	0	0	0	0	3/4	1
Daily living self-efficacy scale^82^	0	0	0	0	1	4	7/12	2
The self-advocacy Scale^93^	0	0	1	1	0	0	6/8	2
Patient self-advocacy Scale^83^	4	0	0	0	0	0	6/10	1
Self-determination Scale^85^	1	0	0	0	1	0	8/10	2
Rosenbaum's Self-control Scale^86^	0	0	3	0	0	0	17/20	1
PROMIS general self-efficacy Scale^84^	0	0	0	0	2	8	0/10	2

All measures covered one, two, or three of the sub-themes. The first four sub-themes were least often covered. In particular, the included self-efficacy measures mostly did not contain items that could be linked to gaining insights, or other aspects conditional to self-regulation. In contrast, for example, the Awareness Questionnaire^89^ covers the sub-themes focussing on gaining insights but none of the other sub-themes. Examples of items covering sub-themes focussing on gaining insights are ‘when I have a health problem, I have a clear understanding’ (sub-theme 1)^91^ and ‘how well can you concentrate now as compared to before your injury?’ (sub-theme 2).^89^

The fifth sub-theme was covered by items from twelve measures, and the sixth sub-theme by items from eight measures. Examples of items are ‘how confident do you feel that you can keep the physical discomfort or pain of your disease from interfering with the things you want to do?’ (sub-theme 5),^24^ ‘I have plans to do enjoyable things for myself’ (sub-theme 6),^91^ ‘take part in new hobbies and new activities’ (sub-theme 6),^24^ and ‘attend an event or go to places on my own’ (sub-theme 6).^24^ None of the measures covered four or more of the sub-themes of self-regulation. For that reason, no evaluation of clinimetric properties was performed.

## Discussion

The purpose of this systematic review was to identify generic measures of self-regulation or related concepts, used in rehabilitation populations, and to analyse the content of these measures. A total of nineteen eligible measures were found throughout the screening process. Content analyses based on our conceptual model of these nineteen measures showed that none of these covered four or more of the six sub-themes of self-regulation considered important in the context of rehabilitation.

Self-regulation is a wide-ranging concept and is seen as a self-learning component of rehabilitation.^6,8,95^ In the current study, we applied a recent broad conceptual model of self-regulation based on subthemes conditional to self-regulation and subthemes on the application of self-regulation in life.^8^ Therefore, it may not come as a surprize that we could not find measures covering all of these subthemes. In our current search, half of the included measures (n = 9) were measures of self-efficacy. Self-efficacy can be described as the confidence persons may have in their abilities to manage their life.^96^ This construct is in line with the fifth subtheme on ‘trust on own body and own functioning’ of self-regulation. Indeed, most of the items of these measures covered the sub-theme of ‘trust in own body and own functioning’. Another related concept was self-awareness. Self-awareness as a construct focusses on the individual's understanding of deficits and the impact of these deficits.^97^ This is in line with the first three sub-themes of the self-regulation model used in this study, which are the sub-themes covered by the Awareness Questionnaire and Self-Awareness subscale.^85^ Lastly, self-control can be defined as ‘informed control over understanding and managing disability or illness’.^81^ In our study the items of self-control items covered mainly the sub-themes on having insight. In conclusion, all these concepts and therewith the measures, either focussed on the sub-themes conditional to regain self-regulation, or on the application of self-regulation.

The systematic review on empowerment measures described also content analyses of the identified measures.^17^ The Health Education Impact Questionnaire^91^ and the Health Locus of Control Scale^67^ were the only measures identified in both studies. The Health Locus of Control Scale was excluded in our study after studying the content of the measure, because the main content of this measure was not directly related to self-regulation. Evaluating the eleven measures in the Empowerment review that were not identified in the current review, these would not have met our inclusion criteria due to a different construct, a different study population, or too specific focus. The Health Education Impact Questionnaire seemed relevant in both reviews, however it did not cover all six sub-themes of the self-regulation model, and therefore we could not conclude this measure would be most appropriate.

It could be discussed whether a measure for self-regulation should include all subthemes. Looking into the literature, all subthemes are found to be important for rehabilitation outcomes to some extent. A study among patients with brain injury proved a focus on the application of self-regulation and higher levels of trust in self, to be effective on rehabilitation outcomes.^98^ Insight in health condition was associated to better quality of life, both on mental and physical health, and to satisfaction with participation.^13,99,100^ Further, awareness of capabilities and illness perception were associated to higher levels of HRQoL and participation.^101,102^ Also, in a study on COPD was found that not just application themes of self-regulation are found to be important, but also the conditional aspects, such as self-insight, are important for outcome performance.^103^ Studies on other rehabilitation population substantiate this statement.^104,105^ With this having discussed, it can be stated that indicators for successful interventions and better clinical rehabilitation outcomes could be found in the combination of conditional as well as application aspects of self-regulation.^106^

Three options to move forward towards comprehensive measurement of self-regulation could be discussed. First, multiple existing measures could be combined to measure the full concept of self-regulation. However, most of the reviewed measures contain multiple items not fitting any of the subthemes of self-regulation. Combining measures would further lead to a long list of items that will be time-consuming and burdening to complete. The second option is to select one existing measure, and accept that the full concept of self-regulation is not covered with that measure. However, from a scientific point of view this is not desirable. The final option is to develop a new measure containing items in line with the conceptual model, without irrelevant items. This would mean less burden for the patient to complete the measure. Beneficial to this option is that the PROM guidelines can be taken into account from the start which guarantees good quality. Items from the existing measures could be used.

The strength of this review was the comprehensive search for measures that met underlying sub-themes of the process to enable self-regulation, based on patients’ perspectives, which was not done earlier. Limitations of this study include the risk for publication bias since the review was focussed only on English language publications and measures, e.g. a self-determination measure was excluded because it was only available in Arabic.^36^ Further, we did not include validation articles and therefore we might have missed measures that were validated but not used in rehabilitation research. To check whether we missed potentially eligible measures because of this, we revisited all excluded validation articles. Only two measures looked potentially eligible for inclusion but they would have been excluded because these focussed on the injury itself or on health care.^107,108^ Other measures were already included or excluded for other reasons. Another limitation might be the subjective nature of the systematic review. First, we did the selection of related concepts of self-regulation, based upon literature, definitions and own perceptions. Further, we categorised the items among the sub-themes based upon our own perspectives. In order to make this more objective, we double checked and high levels of interrater agreement were assured before continuing. If there were any doubts, discussions were held between all four researchers until consensus was obtained. Also, related concepts of self-regulation were selected with the help of an independent research assistant and an information specialist from the library. Lastly, we searched for articles published since January 1^st^ 2015, so we might have missed eligible measures not used in recent years. Further research could be performed in the development of a generic measure based on the conceptual model for self-regulation.

There is a large number of measures developed in health care to measure self-regulation or relating concepts. Much of these measures are however developed for use in a specific diagnostic group or focussed on one specific topic. The present study provides an overview of generic self-regulation measures used between 2015 and mid-2020 in a former rehabilitation population. Development of a comprehensive generic measure of self-regulation could be used to validate the conceptual model and for the understanding of self-regulation in individuals of a non-Dutch rehabilitation population.

**Clinical messages**

- Measurement of conditional and application subthemes of self-regulation is important to optimise clinical rehabilitation outcomes.
- Several measures were identified in this study but none covered all subthemes of self-regulation.
- To measure the whole construct of self-regulation a generic measure should be developed.

## Supplemental Material

sj-docx-1-cre-10.1177_02692155221091510 - Supplemental material for Measures of self-regulation used in adult rehabilitation populations: A systematic review and content screening Click here for additional data file. Supplemental material, sj-docx-1-cre-10.1177_02692155221091510 for Measures of self-regulation used in adult rehabilitation populations: A systematic review and content screening by T.I. Mol, C.A.M. van Bennekom, E.W.M. Scholten, and M.W.M. Post in Clinical Rehabilitation

## Appendix

**Appendix 1.** Excluded measures.

Measurement instrument (Author: year)	A^a^	B^b^	C^c^	D^d^	E^e^	F^f^
1. The self-efficacy scale (Lorig & Ritter)^24^	X					
2. Chronic disease self-efficacy scale (Lorig & Ritter)^24^	X					
3. Marcus self-efficacy for physical activity scale (Marcus)^25^	X					
4. Decision self-efficacy scale (O’Connor)^26^	X					
5. Self-management of own behaviour questionnaire (Lorig & Ritter)^24^	X					
6. General self-efficacy item bank (PROMIS)^27^	X					
7. Pearling-schooler mastery Scale (Pearling & Schooler)^28^	X					
8. Disability management self-efficacy scale (Ammtman)^29^	X					
9. The general competence scale (Dutch version of General SES)^88^	X					
10. Self-efficacy questionnaire (Takasaki)^31^	X					
11. Tennessee self-concept scale (Marsh & Richards)^32^		X				
12. Self-regulatory efficacy scale (Davis et al.)^33^		X				
13. Behaviour rating of executive functions-Adults (Ciszewski et al.)^39^		X				
14. Sociotropy autonomy scale (Beck & Bieling)^34^		X				
15. Five factor self-concept questionnaire (Baer et al.)^35^		X				
16. Self-developed self-determination scale (Al’zboon)^36^		X				
17. Self-constructed self-efficacy questionnaire (Cooper)^38^		X				
18. Self-concept scale using Roys adaption model (Azarmi & Farsi)^37^		X				
19. Multidimensional health locus of control questionnaire (Wallston)^67^			X			
20. Perceived deficits questionnaire (Takasaki)^31^			X			
21. Patient competency rating scale (Kolakowsky et al.)^68^			X			
22. (Revised) Illness perception questionnaire (Moss-Morris)^69^			X			
23. Five facet mindfulness questionnaire (Baer et al.)^35^			X			
24. Enfranchisement scale (Heinemann et al.)^70^			X			
25. The mastery scale (Pearlin & Schooler)^28^			X			
26. The automatic regulation scale (Kroz)^71^			X			
27. The rey auditory verbal learning test (Rey)^72^			X			
28. The mindfulness self-efficacy scale (Cayoun)^73^			X			
29. Karnofsky performance status scale (Schag)^64^			X			
30. Patient global impression on change (Guy)^66^			X			
31. The treatment self-regulation questionnaire (Williams et al.)^65^			X			
32. Moorong self-efficacy scale (Middleton)^40^				X		
33. The self-efficacy questionnaire of Marcus (Marcus)^25^				X		
34. The self-regulation Inventory- Scale (Marques)^51^				X		
35. Control preferences scale (Degner)^56^				X		
36. The self-efficacy for symptom management scale (Cicerone & Azulay)^57^				X		
37. Patient competence questionnaire (Aderhold)^58^				X		
38. Self-efficacy for functional ability scale (Liu et al.)^59^				X		
39. 12-item Physical activity self-regulation scale (Umstattd et al.)^60^				X		
40. The health self-efficacy scale (Lee et al.)^61^				X		
41. The index of self-regulation scale (Yeom et al.)^62^				X		
42. The maintain function scale (Fors et al.)^63^				X		
43. Goal self-efficacy instrument (Karoly & Ruehlman)^41^				X		
44. Situational self-efficacy scale (Rumrill)^42^				X		
45. Self-developed self-determination questionnaire (Paech & Lippke)^43^				X		
46. The perceived competence scale (Williams, Freedman & Deci)^44^				X		
47. The recovery self-efficacy scale (Luszczynska & Sutton)^45^				X		
48. Norman self-efficacy scale (Norman)^46^				X		
49. Self-regulation questionnaire (Carey)^47^				X		
50. Task self-efficacy scale (Blanchard et al.)^48^				X		
51. Patient self-efficacy questionnaire (Lim et al.)^49^				X		
52. Continuity and discontinuity of self-scale (Salander)^50^				X		
53. Self-regulation questions (Martin-Ginis)^52^				X		
54. Self-efficacy for rehabilitation outcome scale (Waldrop et al.)^53^				X		
55. Boston university empowerment scale (Rogers et al.)^54^				X		
56. Self-efficacy for performing energy conservation strategies assessment (Liepold & Mathiowetz)^55^				X		
57. Self-awareness of deficits interview (Malouf)^74^					X	
58. Self-regulation skills interview (Ownsworth)^75^					X	
59. Self-efficacy for core competences (Bender et al.)^76^					X	
60. Self-constructed scale on theory of planned behaviour (Doherty et al.)^77^						X
61. Self-developed self-efficacy to guide behaviour change scale (Nessen et al.)^78^						X

*Category:.

^a^ A: Duplicates. These measures turned out duplicates used under a different name.

^b^ B: Not available (also not after request from author). These measures were mentioned throughout the studies, however the measure was not free available to screen the items (content) of the measure.

^c^ C: Measuring another construct. These measures turned out to measure a construct which was not related to self-regulation: meaning a concept which is not included in the search string.

^d^ D: Too specific. These measures were excluded due to the fact they were not generic. This means these measures were specifically developed and adapted to a specific diagnosis such as spinal cord injury, or the measure was focussed on one specific domain of life such as self-regulation in physical activity.

^e^ E: No questionnaire. These measures turned out to be an interview, not a PROM.

^f^ F: Wrong inclusion. These measures had to be excluded based on the inclusion criteria in the prior phase, however they were overlooked and excluded in this phase due to reasons such as ‘other target group’.
