# Supplementary material for: Measures of self-regulation used in adult rehabilitation populations: A systematic review and content screening
Source: Clin Rehabil. 2022 Apr 27;36(8):1120–38. doi: 10.1177/02692155221091510 (PMC9284405; doi:10.1177/02692155221091510)
Supplement: sj-docx-1-cre-10.1177_02692155221091510 - Supplemental material for Measures of self-regulation used in adult rehabilitation populations: A systematic review and content screening [file sj-docx-1-cre-10.1177_02692155221091510.docx]

**ONLINE SUPPLEMENT: Search strings**

**Pubmed**

| **Search** | **Construct** | **Query** |
| --- | --- | --- |
| #1 | Construct: Self-regulation (and related terms) | (("Self Concept"[Mesh:NoExp]) OR "Self Efficacy"[Mesh:NoExp]) OR "self-concept"[tiab] OR "selfconcept"[tiab] OR "self-control"[MeSH] OR "self-control"[tiab] OR "selfcontrol"[tiab] OR "self-regulat*"[tiab] OR "selfregulat*"[tiab] OR "self-effica*"[tiab] OR "selfeffica*"[tiab] OR "patient advocacy"[MesH] OR "patient advoca*"[tiab] OR "patientadvoca*"[tiab] OR "self-advoca*"[tiab] OR "selfadvoca*"[tiab] OR "patient agency"[tiab] OR "personal agency"[tiab] OR "empowerment"[MeSH] OR "empower*"[tiab] OR “awareness”[tiab] OR “selfawareness”[tiab] OR "personal autonomy"[MeSH] OR "personal autonomy"[tiab] OR "Selfdeterminat*"[tiab] OR "Self-determinat*"[tiab] |
| #2 | Type of instrument: PROM | ("Patient Outcome Assessment"[Mesh] OR patient outcome assessment*[tiab] OR patient centered outcome*[tiab] OR patient reported outcome*[tiab] OR prom[tiab] OR proms[tiab] OR pro[tiab] OR ((patient[tiab] OR self[tiab] OR career[tiab] OR proxy[tiab]) AND ((report[tiab] OR reported[tiab] OR reporting[tiab]) OR (rated[tiab] OR rating[tiab] OR ratings[tiab]) OR based[tiab] OR (assessed[tiab] OR assessment[tiab] OR assessments[tiab] OR assessing[tiab]))) OR ((disability[tiab] OR function[tiab] OR functional[tiab] OR functions[tiab] OR subjective[tiab] OR utility[tiab] OR utilities[tiab] OR wellbeing[tiab] OR well being[tiab]) AND (outcome[tiab] OR outcomes[tiab] OR index[tiab] OR indices[tiab] OR instrument[tiab] OR instruments[tiab] OR measure[tiab] OR measures[tiab] OR questionnaire[tiab] OR questionnaires[tiab] OR profile[tiab] OR profiles[tiab] OR scale[tiab] OR scales[tiab] OR score[tiab] OR scores[tiab] OR status[tiab] OR survey[tiab] OR surveys[tiab]))) |
| #3 | Population: Adults with impairment or chronic disability | (((stroke[tiab] OR spinal cord*[tiab] OR amput*[tiab] OR acquired brain* [tiab] OR chronic pain[tiab] OR oncol*[tiab] OR muskuloskeletal* [tiab] OR neuromusc*[tiab] ) OR (spinal injur*[Title/Abstract])) OR (brain injur*[Title/Abstract) OR (neurolog*[Title/Abstract] ) OR (rehab*) OR (ABI[Title/Abstract]) |
| #4 | NOT | (“address”[Publication Type] OR “biography”[Publication Type] OR “case reports”[Publication Type] OR “comment”[Publication Type] OR “directory”[Publication Type] OR “editorial”[Publication Type] OR “festschrift”[Publication Type] OR “interview”[Publication Type] OR “lecture”[Publication Type] OR “legal case”[Publication Type] OR “legislation”[Publication Type] OR “letter”[Publication Type] OR “news”[Publication Type] OR “newspaper article”[Publication Type] OR “patient education handout”[Publication Type] OR “popular work”[Publication Type] OR “congress”[Publication Type] OR “consensus development conference”[Publication Type] OR “consensus development conference, nih”[Publication Type] OR “practice guideline”[Publication Type]) NOT (“animals”[MeSH Terms] NOT “humans”[MeSH Terms]) |
|  | Filter | 2015 - 2020 |
|  | Filter | Language: English |
| Total |  | (#1 AND #2 AND #3) NOT #4 |

**PsychInfo**

| **Search** | **Construct** | **Query** |
| --- | --- | --- |
| #1 | Construct: Self-regulation (and related terms) | exp Self-Concept/ or self-concept.ti,ab,id. or exp Self-Efficacy/ or self-effica*.ti,ab,id. or exp Self-Regulation/ or self-regulat*.ti,ab,id. or exp Self-Advocacy/ or self-advoca*.ti,ab,id. or patient agency.ti,ab,id. or personal agency.ti,ab,id. or exp Empowerment/ or empowerment.ti,ab,id. or exp Self-Determination/ or self-determinat*.ti,ab,id. or exp Self-Control/ or self-control.ti,ab,id. or exp "independence (personality)"/ or personal autonomy.ti,ab,id. |
| #2 | Type of instrument: PROM  767,283 | exp patient reported outcome measures/ or patient outcome assessment*.ti,ab,id. or patient centered outcome*.ti,ab,id. or patient reported outcome*.ti,ab,id. or prom.ti,ab,id. or proms.ti,ab,id. or pro.ti,ab,id. or ((patient or self or career or proxy) and (report or reported or reporting or (rated or rating or ratings) or based or (assessed or assessment or assessments))).ti,ab,id. or ((disability or function or functional or functions or subjective or utility or utilities or wellbeing or well being) and (outcome or outcomes or index or indices or instrument or instruments or measure or measures or questionnaire or questionnaires or profile or profiles or scale or scales or score or scores or status or survey or surveys)).ti,ab,id. or assessing.ti,ab,id. |
| #3 | Population: Adults with impairment or chronic disability | **Cerebrovascular*.ti,ab. or stroke.ti,ab. or ABI.ti,ab. or spinal cord*.ti,ab. or amput*.ti,ab. or acquired brain* .ti,ab. or chronic pain.ti,ab. or oncol*.ti,ab. or muskuloskeletal* .ti,ab. or neuromusc*.ti,ab. or spinal injur*.ti,ab. or brain injur*.ti,ab. or neurolog*.ti,ab. or exp rehabilitation/ or rehab*.af.** |
| #4 | NOT | 'addresses'.pt. OR 'biography'.pt. OR 'case reports'.pt. OR 'comment'.pt. OR 'directory'.pt. OR 'editorial'.pt. OR 'festschrift'.pt. OR 'interview'.pt. OR 'lectures'.pt. OR 'legal cases'.pt. OR 'legislation'.pt. OR 'letter'.pt. OR 'news'.pt. OR 'newspaper article'.pt. OR 'patient education handout'.pt. OR 'popular works'.pt. OR 'congresses'.pt. OR 'consensus development conference'.pt. OR 'consensus development conference, nih'.pt. OR 'practice guideline'.pt. NOT ('animals'.sh. NOT 'humans'.sh.) |
|  | Filter | 2015 - 2020 |
|  | Filter | Language: English |
| Total |  | (#1 AND #2 AND #3) NOT #4 |

**Embase**

| **Search** | **Construct** | **Query** |
| --- | --- | --- |
| #1 | Construct: Self-regulation (and related terms) | 'self concept'/exp OR 'self concept':ab,ti,kw OR 'self effic*':ab,ti,kw OR 'autoregulation'/exp OR 'autoregulat*':ab,ti,kw OR 'self regulat*':ab,ti,kw OR 'self advocacy'/exp OR 'self advoca*':ab,ti,kw OR 'agency for healthcare research and quality'/exp OR 'agency for healthcare research and quality':ab,ti,kw OR 'patient agency':ab,ti,kw OR 'personal agency':ab,ti,kw OR 'empowerment'/exp OR 'empowerment':ab,ti,kw OR 'self determination'/exp OR 'self determinat*':ab,ti,kw OR 'self control'/exp OR 'self control':ab,ti,kw OR 'personal autonomy'/exp OR 'personal autonomy':ab,ti,kw |
| #2 | Type of instrument: PROM | 'pro':ab,ti OR ('patient':ab,ti,kw OR 'self':ab,ti,kw AND ('report':ab,ti OR 'reported':ab,ti OR 'reporting':ab,ti OR 'rated':ab,ti OR 'rating':ab,ti OR 'ratings':ab,ti OR 'based':ab,ti OR 'assessed':ab,ti OR 'assessment':ab,ti OR 'assessments':ab,ti)) OR ('disability':ab,ti OR 'function':ab,ti,kw OR 'functional':ab,ti OR 'functions':ab,ti OR 'subjective':ab,ti OR 'utility':ab,ti OR 'utilities':ab,ti AND ('outcome':ab,ti OR 'outcomes':ab,ti OR 'index':ab,ti OR 'indices':ab,ti OR 'instrument':ab,ti OR 'instruments':ab,ti OR 'measure':ab,ti OR 'measures':ab,ti OR 'questionnaire':ab,ti OR 'questionnaires':ab,ti OR 'profile':ab,ti OR 'profiles':ab,ti OR 'scale':ab,ti OR 'scales':ab,ti OR 'score':ab,ti OR 'scores':ab,ti OR 'status':ab,ti OR 'survey':ab,ti OR 'surveys':ab,ti)) |
| #3 | Population: Adults with impairment or chronic disability | **'cerebrovasc*':ab,ti,kw OR ‘stroke’:ab,ti,kw** OR ‘ABI’:ab,ti,kw **OR ‘spinal cord*’:ab,ti,kw OR ‘amput*’:ab,ti,kw** OR ‘acquired brain*’:ab,ti,kw OR ‘chronic pain’:ab,ti,kw OR ‘oncol*’:ab,ti,kw OR ‘muskuloskeletal*’:ab,ti,kw OR ‘neuromusc*’:ab,ti,kw OR ‘spine injur*’:ab,ti,kw OR ‘brain injur*’:ab,ti,kw OR ‘neurolog*’:ab,ti,kw OR 'rehabilitation':lnk OR ‘rehab*’ |
| #4 | NOT | ‘addresses’:it OR ‘biography’:it OR ‘case reports’:it OR ‘comment’:it OR ‘directory’:it OR ‘editorial’:it OR ‘festschrift’:it OR ‘interview’:it OR ‘lecture’:it OR ‘legal case’:it OR ‘legislation’:it OR ‘letter’:it OR ‘news’:it OR ‘newspaper article’:it OR ‘patient education handout’:it OR ‘popular work’:it OR ‘congress’:it OR ‘consensus development conference’:it OR ‘consensus development conference, nih’:it OR ‘practice guideline’:it NOT ('animals'/exp NOT 'humans'/exp) |
| Filter |  | 2015 - 2020 |
| Filter |  | Language: English |
| Total |  | (#1 AND #2 AND #3) NOT #4 |

**CINAHL**

| **Search** | **Construct** | **Query** |
| --- | --- | --- |
| #1 | Construct: Self-regulation (and related terms) | (MH "Self-Efficacy+") OR (TI "Self-Effic*") OR (AB "Self-Effic*") OR (MH "Self Concept+") OR (TI "Self Concept") OR (AB "Self Concept") OR (MH "Self Regulation+") OR (TI "Self Regulat*") OR (AB "Self Regula*") OR (MH "Self-Advocacy+") OR (TI "Self-Advoca*") OR (AB "Self-Advoca*") OR (MH "Self Care Agency+") OR (TI "Self Care Agency") OR (AB "Self Care Agency") OR (TI "personal agency") OR (AB "personal agency") OR (TI "patient agency") OR (AB "patient agency") OR (MH "Empowerment+") OR (TI "Empowerment") OR (AB "Empowerment") OR (MH "Patient Autonomy+") OR (TI "Patient Autonomy") OR (AB "Patient Autonomy") OR (TI "self determinat*”) OR (AB "self determinat*”) OR (MH "Control (Psychology)+") OR (TI "Control (Psychology)") OR (AB "Control (Psychology)") OR (TI "self control") OR (AB "self control") |
| #2 | Type of instrument: PROM | (MM "Outcome Assessment") OR (MM "Patient Assessment+") OR (MM "Patient-Reported Outcomes") OR (TI “patient outcome assessment*”) OR (TI “patient centered outcome*”) OR ( TI “patient reported outcome*”) OR (TI “prom”) OR (TI “proms”) OR (TI “pro”) OR ((patient”)OR (TI “self”) OR (TI “career”) OR (TI “proxy”)) AND ((report”) OR (TI “reported”) OR (TI “reporting”)) OR (TI “(rated”) OR (TI “rating”) OR (TI “ratings”)) OR (TI “based”) OR (TI “(assessed”) OR (TI “assessment”) OR (TI “assessments”)))) OR ((disability”) OR (TI “function”) OR (TI “functional”) OR (TI “functions”) OR (TI “subjective”) OR (TI “utility”) OR (TI “utilities”) OR (TI “wellbeing”) OR (TI “well being”)) AND ( TI “outcome”) OR (TI “outcomes”) OR (TI “index”) OR (TI “indices”) OR (TI “instrument”) OR (TI “instruments”) OR (TI “measure”) OR (TI “measures”) OR (TI “questionnaire”) OR (TI “questionnaires”) OR (TI “profile”) OR (TI “profiles”) OR (TI “scale”) OR (TI “scales”) OR (TI “score”) OR (TI “scores”) OR (TI “status”) OR (TI “survey”) OR (TI “surveys”) ))) OR (TI “assessing”) OR (AB “patient outcome assessment*”) OR (AB “patient centered outcome*”) OR ( AB “patient reported outcome*”) OR (AB “prom”) OR (AB “proms”) OR (AB “pro”) OR ((patient”)OR (AB “self”) OR (AB “career”) OR (AB “proxy”)) AND ((report”) OR (AB “reported”) OR (AB “reporting”)) OR (AB “(rated”) OR (AB “rating”) OR (AB “ratings”)) OR (AB “based”) OR (AB “(assessed”) OR (AB “assessment”) OR (AB “assessments”)))) OR ((disability”) OR (AB “function”) OR (AB “functional”) OR (AB “functions”) OR (AB “subjective”) OR (AB “utility”) OR (AB “utilities”) OR (AB “wellbeing”) OR (AB “well being”)) AND ( AB “outcome”) OR (AB “outcomes”) OR (AB “index”) OR (AB “indices”) OR (AB “instrument”) OR (AB “instruments”) OR (AB “measure”) OR (AB “measures”) OR (AB “questionnaire”) OR (AB “questionnaires”) OR (AB “profile”) OR (AB “profiles”) OR (AB “scale”) OR (AB “scales”) OR (AB “score”) OR (AB “scores”) OR (AB “status”) OR (AB “survey”) OR (AB “surveys”) ))) OR (AB “assessing”) |
| #3 | Population: Adults with impairment or chronic disability | (TI “Cerebrovascular*“) OR (AB “Cerebrovascular*“) OR (TI “stroke“) OR (AB “stroke“) OR (TI “ABI“) OR (AB “ABI“) OR (TI “spinal cord*“) OR (AB “spinal cord*“) OR (TI “amput*“) OR (AB “amput*“) OR (TI “acquired brain*“) OR (AB “acquired brain*“) OR (TI “chronic pain“) OR (AB “chronic pain“) OR (TI “oncol*“) OR (AB “oncol*“) OR (TI “muskuloskeletal*“) OR (AB “muskuloskeletal*“) OR (TI “neuromusc*“) OR (AB “neuromusc*“) OR (TI “spinal injur*“) OR (AB “spinal injur*“) OR (TI “brain injur*“) OR (AB “brain injur* “) OR (TI “neurolog*”) OR (AB “neurolog*”) OR (MM "Rehabilitation+") OR “rehab*” |
| #4 | NOT | (PT (“adressess” OR “biography” OR “case reports” OR “comment” OR “directory” OR “editorial” OR “festschrift” OR “interview” OR “lectures” OR “legal cases” OR “legislation” OR “letter” OR “news” OR “newspaper article” OR “patient education handout” OR “popular works” OR “congresses” OR “consensus development conference” OR “consensus development conference, nih” OR “practice guideline”)) NOT (MH (“animals” NOT “humans”)) |
| Filter |  | 2015 - 2020 |
| Filter |  | Language: English |
| Total |  | (#1 AND #2 AND #3) NOT #4 |
